# Implementing a context-driven awareness programme addressing household air pollution and tobacco: a FRESH AIR study

**DOI:** 10.1038/s41533-020-00201-z

**Published:** 2020-10-06

**Authors:** Evelyn A. Brakema, Frederik A. van Gemert, Sian Williams, Talant Sooronbaev, Berik Emilov, Maamed Mademilov, Aizhamal Tabyshova, Pham Le An, Nguyen Nhat Quynh, Le Huynh Thi Cam Hong, Tran Ngoc Dang, Rianne M. J. J. van der Kleij, Niels H. Chavannes, Corina de Jong, The FRESH AIR collaborators, Marilena Anastasaki, Azamat Akylbekov, Andy Barton, Antonios Bertsias, Pham Duong Uyen Binh, Job F. M. van Boven, Dennis Burges, Lucy Cartwright, Vasiliki E. Chatzea, Liza Cragg, Ilyas Dautov, Irene Ferarrio, Ben Hedrick, Nick Hopkinson, Elvira Isaeva, Rupert Jones, Sanne van Kampen, Winceslaus Katagira, Jesper Kjærgaard, Janwillem Kocks, Le Thi Tuyet Lan, Tran Thanh Duv Linh, Christos Lionis, Kim Xuan Loan, Andy McEwen, Patrick Musinguzi, Rebecca Nantanda, Grace Ndeezi, Sophia Papadakis, Hilary Pinnock, Jillian Pooler, Charlotte C. Poot, Maarten J. Postma, Anja Poulsen, Pippa Powell, Susanne Reventlow, Dimitra Sifaki-Pistolla, Sally Singh, Jaime Correia de Sousa, James Stout, Marianne Stubbe Østergaard, Ioanna Tsiligianni, Tran Diep Tuan, James Tumwine, Le Thanh Van, Nguyen Nhu Vinh, Simon Walusimbi, Louise Warren

**Affiliations:** 1 Department of Public Health and Primary Care, Leiden University Medical Center, Leiden, The Netherlands; 2 Groningen Research Institute for Asthma and COPD (GRIAC) & Department of General Practice & Elderly Care Medicine, University of Groningen, University Medical Center Groningen, Groningen, The Netherlands; 3 Makerere University Lung Institute (MLI), Mulago Hospital, Kampala, Uganda; 4 International Primary Care Respiratory Group (IPCRG), London, UK; 5 Pulmonary Department, National Center of Cardiology and Internal Medicine, Bishkek, Kyrgyzstan; 6 Center of Training Family Medicine, University of Medicine and Pharmacy, Ho Chi Minh City, Vietnam; 7 Clinic of Social and Family Medicine, School of Medicine, University of Crete, Heraklion, Greece; 8 Faculty of Medicine and Dentistry, University of Plymouth, Plymouth, UK; 9 University of Groningen, University Medical Center Groningen, Groningen, The Netherlands; 10 Department of Pediatrics, University of Washington School of Medicine, Seattle, WA USA; 11 European Lung Foundation, Sheffield, UK; 12 Imperial College London, London, UK; 13 The Research Unit for General Practice and Section of General Practice, Department of Public Health, Copenhagen University, Copenhagen, Denmark; 14 Global Health Unit, The Department of Paediatrics and Adolescent Health, Juliane Marie Center, Copenhagen University Hospital “Rigshospitalet”, Copenhagen, Denmark; 15 National Centre for Smoking Cessation and Training, Dorchester, UK; 16 University of Edinburgh, Usher Institute of Population Health Sciences and Informatics, Edinburgh, UK; 17 Coventry University, Coventry, UK; 18 University of Minho, School of Medicine, Braga, Portugal

**Keywords:** Disease prevention, Patient education, Respiratory tract diseases, Public health, Translational research

## Abstract

Most patients with chronic respiratory disease live in low-resource settings, where evidence is scarcest. In Kyrgyzstan and Vietnam, we studied the implementation of a Ugandan programme empowering communities to take action against biomass and tobacco smoke. Together with local stakeholders, we co-created a train-the-trainer implementation design and integrated the programme into existing local health infrastructures. Feasibility and acceptability, evaluated by the *modified Conceptual Framework for Implementation Fidelity*, were high: we reached ~15,000 Kyrgyz and ~10,000 Vietnamese citizens within budget (~€11,000/country). The right engaged stakeholders, high compatibility with local contexts and flexibility facilitated programme success. Scores on lung health awareness questionnaires increased significantly to an excellent level among all target groups. Behaviour change was moderately successful in Vietnam and highly successful in Kyrgyzstan. We conclude that contextualising the awareness programme to diverse low-resource settings can be feasible, acceptable and effective, and increase its sustainability. This paper provides guidance to translate lung health interventions to new contexts globally.

## Introduction

Chronic respiratory diseases (CRDs) are a major burden to health worldwide, with chronic obstructive pulmonary disease (COPD) being the third leading cause of death^1^. The vast majority of deaths related to CRD occur in low- and middle-income countries (LMICs)^2–4^. While the prevalence of major risk factors to CRD—smoking and household air pollution (HAP)—is commonly high in LMICs, means to combat the risks are low^5–10^. Preventing CRD is the most affordable and effective strategy for decreasing the burden^4^. This would involve solutions such as smoking cessation and providing alternatives for cooking and heating on solid fuels in poorly ventilated homes. However, for decades, implementation of such interventions in local communities has demonstrated to be challenging^11–14^.

An important reason for implementation failure is the misalignment of local knowledge and beliefs with the interventions offered and their implementation strategies^15–19^. If there is no locally perceived need for change, motivation for behaviour change is low^20,21^. Particularly in rural areas of LMICs, awareness about CRDs and the risks of tobacco and biomass fuel smoke is low. COPD as a disease, and the implications of asthma, are often unknown to local community members, policy makers and health workers^4,22^. This affects the quality of care and prevents communities from taking simple steps to avoid smoke exposure^5,23–27^. In addition, the use of biomass fuels is determined by poverty^28,29^. Motivating low-income household to purchase cleaner stoves and fuels is generally beyond their means^28,30,31^. Therefore, for successfully reducing risk behaviour, preventive interventions are needed that understand and address these barriers to behaviour change.

An intervention to raise awareness about CRDs and empower communities with realistic measures to reduce exposure to risk factors was conducted in Uganda^32^. The programme was underpinned by the capability, opportunity, motivation—behaviour (COM-B) model. Changing behaviour of individuals, groups or populations involves addressing one or more of the COM elements^33^. By raising knowledge and awareness of CRD and the harms of smoke exposure (capability) and providing realistic, affordable solutions to prevent exposure (opportunity), participants were stimulated (motivation) to reduce risk behaviour (behaviour). This awareness programme had a cascading train-the-trainer structure and started with healthcare workers (HCWs) with medical knowledge, who then trained community health workers (CHWs) with limited medical knowledge, who trained their communities. CHWs were considered the key players in raising awareness. They are chosen from their own community and play a crucial role in providing primary healthcare in low-resource settings; often, they are the only ones available to provide direct medical assistance in their community^34,35^. The programme demonstrated to be feasible, acceptable and effective^32^. Potentially, this programme could be widely applicable to other settings across the world.

However, effectively translating evidence-based interventions to other settings is considered by the World Health Organization (WHO) as among the biggest challenges of the twenty-first century^36^. Failure to adequately translate and implement interventions can seriously comprise their effectiveness^37,38^. Practical guidance on how to translate a preventive programme addressing awareness on CRD and empowering communities to change risk behaviour is unavailable. Therefore, our aim was to study the feasibility, acceptability and effectiveness of translating an awareness programme targeting risks to CRD to two completely different contexts in Kyrgyzstan and Vietnam and provide lessons learned from this process.

## Results

Details on the awareness programme and the deployed implementation strategy are provided in Box 1. A structured evaluation of the programme’s feasibility, acceptability and fidelity is detailed in Table 1.

**Table 1:** Implementation fidelity of the awareness programme.

Elements of fidelity	Kyrgyzstan	Vietnam
Adherence (was the programme implemented as it was designed?)		
Content	We used the session content template addressing elements of the COM-B model (Appendix 3) in each training of HCWs in workstream 1. A concise version was used for the training of the health workers in workstream (2 and) 3	
	The content displayed on flip-overs and posters (Appendix 4) was aligned with the session content template	
		A tradition of constantly burning coal around a new-born during 1 month turned out to be also relevant, but was not addressed
Coverage	Direct reach: 10 HCWs were trained first. We had planned to train 50 health workers from different levels (e.g. CHWs and social workers). Due to high enthusiasm of trained health workers, we trained 90. Trained health workers reported to have been in contact with 80–160 community members each month, training ~15,000 community members within 6 months	Direct reach: 17 HCWs were trained first (one per health centre). Each centre covered 3–7 villages, resulting in 77 trained CHWs. Each CHW reported to have contact with 100–150 community members and so reached ~10,000 community members directly within 6 months
	Number of drop-outs was not registered	
Frequency/duration	Initial training was 2 days shorter than in Uganda, due to experience facilitating the training in Uganda and because the materials were in a further development stage	
	Initial group of HCWs was trained for 3 days, CHWs and social workers were then trained for half a day within 3 months after HCW training	Initial group of HCWs was trained for 3 days, new group of HCWs trained for 1 day within 3 months and CHWs trained for half a day within another 3 months
	Outside of the programme, the training was used to train HCWs from neighbouring countries during an international conference (IPCRG in Bishkek, 2018)	
	Training of communities is ongoing to date. Using the materials, training continues to take place to patients and their families during visits to health facilities	
Moderators (factors that have influenced the degree of fidelity)		
Intervention complexity	Simplicity was enhanced by accompanying the training materials with short, explicit explanations and illustrations, e.g. specific instructions on the back of flip-overs with main messages to be addressed	
	The module and training materials were translated in the local languages	
	We co-created training materials together with health workers and other stakeholders to ensure easy understanding	
Facilitation strategy	We strategically engaged stakeholders through collaboration meetings and hence enhanced (1) compatibility with the local context by co-developing the delivery strategy with them and (2) continuation of the programme through their support and ownership of the programme	
	We adapted the strategy and programme materials to the local settings in collaboration with local stakeholders, HCWs, CHWs and the community. Key messages remained identical	
	An active session was held on the national state TV channel, supplemented by messages on the radio and newspapers	The budget for a media campaign was exchanged for refresher courses of the trainers
Quality of delivery	HCWs and CHWs were trained on how to train. Training was supported by materials: both local FRESH AIR teams chose to use a PowerPoint for the health workers training, flip-overs for training the community, …	
	… and brochures + posters to be distributed to health centres/public spaces	… and printed flip-overs instead of posters as the budget did not allow for printing additional brochures. The local team also delivered refresher courses for monitoring and feedback
Participant responsiveness	HCWs and CHWs reported and demonstrated to feel ownership due to the co-creating process. The enthusiastic participation of communities and observed behaviour change (e.g. adoption of changed cooking practices) motivated the health workers to continue the process	
	CHWs (and social workers) reported high numbers of community members reached, which was confirmed by triangulation with the number of collected knowledge questionnaires	
Recruitment	All participants were recruited within the existing health infrastructure	
	District health managers with expert knowledge on the local context selected the first HCWs to be trained	
	Some of our research team members participated in this first group of HCWs. The local FRESH AIR team explained that they were more easily available than regular HCWs to travel (which took relatively long in Kyrgyzstan due to the rough terrains). Also for the sake of travel time, these HCWs trained other health workers (CHWs and social workers) directly instead of via workstream 2 (Fig. 1)	One HCW per ward (the head of the health station) was selected for the initial training. They selected the next group based on convenience
	CHWs were purposely selected based on convenience (living in villages in vicinity of health centres), in collaboration with local HCWs	
	Community members were recruited during regular health events	CHWs and the local team organised health sessions
Context	Local context was well known due to preliminary explorative FRESH AIR fieldwork, due to close collaboration with the stakeholders and because our team consisted of local and international team members	
		Due to a miscommunication with the local and coordinating team, a costly pilot study was conducted assessing the frequency of biomass fuel use. However, the high frequency of use reassured the relevance to the selected setting
	Compatibility with the local context was enhanced by adapting interventions in collaboration with local stakeholders, HCWs and CHWs and by embedding the intervention within the local healthcare system	

*COM-B model* capability, opportunity, motivation—behaviour model, *HCW* healthcare worker, *CHW* community health worker, structured by the modified *Conceptual Framework for Implementation Fidelity*.

Box 1 The awareness programme and its implementation strategy **The awareness programme**

The programme aimed to increase local knowledge on CRDs and major risk factors (tobacco and biomass smoke) and to empower communities to reduce exposure to the risk factors. This included awareness on feasible and acceptable behavioural change interventions for smoking cessation and second-hand smoke exposure^54^. It also included specific measures to reduce HAP, targeting (1) the source of the smoke (promoting clean fuels and improved stoves), (2) the living environment (improving ventilation and kitchen design), and (3) the user (drying fuel, using pot lids, maintaining the stoves well, keeping small children (and if possible pregnant women) away from the smoke and cooking outdoors)^64^.

**Fig. 1:**
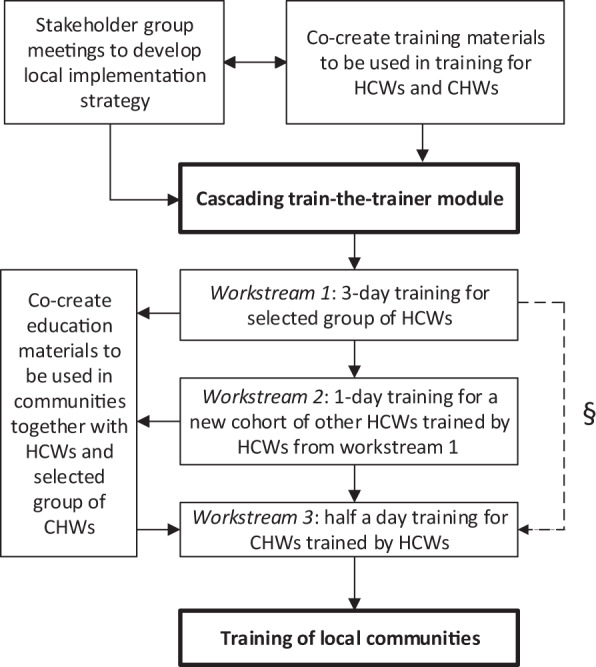
Design of the awareness programme. HCW healthcare worker, CHW community health workers. ^§^Workstream 2 is optional.

The programme followed a cascading train-the-trainer approach: HCWs first received an intense 3-day training. Besides the content above, the training also included co-creation of programme materials by HCWs and the team and instruction how to use those. Lastly, training skills were addressed, such as different training techniques and methodologies for adult learners and community mobilisation techniques. Next, HCWs trained other HCWs (1 day), who subsequently trained CHWs (half day), who in turn trained their communities (Fig. 1). An overview of the content and how it addressed the COM-B elements is displayed in Supplementary Methods.

**Co-development of the implementation strategy**

Ensuring to embed our programme in the local existing health infrastructure, we co-developed the implementation strategy with local influential and knowledgeable stakeholders (ranging from a popular national artist, to community members, to district health officers (Supplementary Table 1)). During a series of meetings, contextual factors (Supplementary Table 1) were discussed. Together we defined the programme’s exact aim, intended outcomes and delivery method. Stakeholders in both settings endorsed the train-the-trainer implementation strategy and considered the programme outline (Fig. 1) used in Uganda also appropriate for their own setting.

**Co-creation of the training materials**

**Fig. 2:**
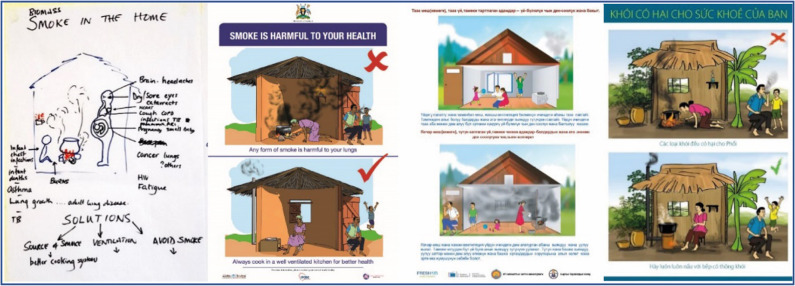
Development of the illustrations, from the first draft (left) to the final version used in Uganda, Kyrgyzstan and Vietnam. The illustrations show solutions to smoke exposure (use of improved stoves, improve ventilation by opening a window or installing a chimney, quit smoking, etc.). Illustrations were made by the art department of local universities.

Training materials included posters for clinics and other public places, flip-over charts for HCWs and CHWs (with pictorial messages for communities and instructions for HCWs and CHWs on the back), brochures and seminar materials (Supplementary Methods). Materials used in Uganda^32^ were first translated to Vietnamese and Russian. Together with the stakeholders, we then contextually adapted the materials to local conditions while maintaining essential elements. For example, we continued to address tobacco but made changes to the type of tobacco smoked. We also adapted the house, skin colour and background (Fig. 2). Illustrations were made by the art department of local universities. The Kyrgyz Ministry of Health and the Vietnamese Center for Health Communication and Education approved the materials for national use.

### Feasibility

The awareness programme was implemented as planned, without delays within the 3-year timeline of the FRESH AIR (*F*ree *R*espiratory *E*valuation and *S*moke-exposure reduction by primary *H*ealth c*A*re *I*ntegrated g*R*oups) project (Table 1). Costs remained within the budgeted €11,000 per setting, although there were local variations (Table 2). For example, travel costs were high in Kyrgyzstan, with rough mountainous terrains. In Vietnam, norms in the health infrastructure prescribed that all additional training time for health workers had to be financially compensated.

**Table 2:** Costs of the awareness programme, compared to Uganda.

		Kyrgyzstan	Vietnam	Uganda
Intervention				
PowerPoint	Translation	700	850	1530
Posters	Translation and printing	1000	n.a.	660
Flip-overs	Translation and printing	700	750	830
Training HCWs		500	1000	3830
Training CHWs		500	1900	1050
Training community		500	3300	0
Travel costs for training		3000	600	360
Media campaign		1000	n.a.	2060
Refresher course		0	1000	680
Planning		0	650	0
Accommodation		3000	0	0
TOTAL		10,900	10,050	11,000
Study activities				
Preparation final report		^a^	400	^a^
Pre- and post-test HCWs		500	^a^	24
Pre- and post-test CHWs		500	350	46
Pre- and post-test community		500	4000	1450
Travel costs pre- and post-test		3000	200	480
TOTAL		4500	4950	2000
Intervention + study				
TOTAL		15,400	15,000	13,000

Costs are in euros.

*HCW* healthcare worker, *CHW* community health worker, *n.a.* not applicable.

^a^Not tracked separately. Note, the pilot study in Vietnam that was conducted due to a miscommunication is not included in this overview.

### Fidelity

Generally, the steps of the programme were adhered to as intended (Fig. 1 and Table 1). We co-developed the local implementation strategy with local stakeholders, co-created the programme’s materials (Fig. 2) and completed a train-the-trainer cascade. We slightly deviated from the planned delivery method in Kyrgyzstan; the relatively long travel times due to rough terrains in Kyrgyzstan resulted in an adapted structure in our cascade.

### Essential components of the implementation strategy

Adequate knowledge of the local context was essential to successful programme implementation. This included knowledge of the health and political infrastructure, to ensure embedment of the programme into it. For example, capitalising on the vital role of CHWs demonstrated to be an effective and sustainable delivery strategy. CHWs were already trusted by communities and trained to deliver knowledge; the programme simply additionally equipped them with relevant medical knowledge to spread. Adequate knowledge of the local context also included knowledge of local beliefs and behaviours regarding respiratory symptoms and risks. For example, a polite Vietnamese habit to invite a male stranger to a conversation is offering him a cigarette. The programme hence needed to address how to join a conversation without having to smoke the cigarette.

We also considered it crucial to collaborate with local authorities, promote community participation and engage local knowledgeable and influential stakeholders (Supplementary Methods). Engaging stakeholders from the beginning enabled us to learn about the local context and also created the sense of ownership needed for sustained use of the programme. Although the bureaucratic approval process of the programme’s materials by national authorities resulted in a delay of several months, this collaboration with local authorities was needed for a sustained implementation.

We did not reach consensus on the necessity to train through a full cascading structure. The local Kyrgyz team believed that omitting workstream 2 (Fig. 1) would increase implementation success, while the coordinating team had the impression that for efficiency and sustainability of the programme, preferably all workstreams should be involved.

Lastly, flexibility was an important component. Many important stakeholders or contextual factors only revealed themselves along the way; the programme and delivery should be highly adaptable to continue to promote compatibility with the context.

### Effectiveness

**Fig. 3:**
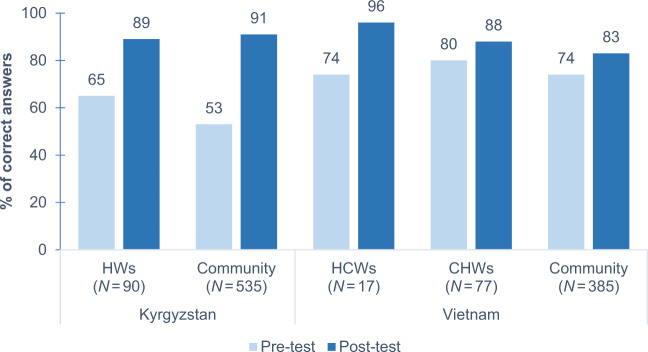
Knowledge questionnaire scores. HW health worker (CHW and social worker), HCW healthcare worker, CHW community health worker. All differences between pre- and post-training scores were significant (*P* < 0.05; Wilcoxon signed-rank tests). In Kyrgyzstan, the ten HCWs were not included, as some members were part of the FRESH AIR team.

On the immediate psychological capability level in the COM-B, the percentage of questions answered correctly on the knowledge questionnaire improved significantly among all groups in both countries (Fig. 3 and Supplementary Results). In Kyrgyzstan, knowledge was initially more limited, but improvements were larger. Notably, in Kyrgyzstan we did not assess the initial group of HCWs as this group included local FRESH AIR team members.

On the longer-term behavioural level, acceptability of the improved stoves was high: 100% of the stove users in Kyrgyzstan and 89.8% in Vietnam recommended the new stove to others. Stove stacking occurred in 15% of the Kyrgyz households and 85.5% of the Vietnamese^39^. In Vietnam, the improved cookstoves were often considered too small: 44% continued to use the traditional cookstove for cooking every day and 36% for several times a week.

## Discussion

In this study, we translated an awareness programme on the risks of biomass fuel and tobacco smoke to lung health, proven effective in Uganda, to two completely different low-resource settings: Kyrgyzstan and Vietnam. We demonstrated that the implementation of the programme was highly feasible and acceptable in both new settings. It was highly effective in Kyrgyzstan and moderately effective in Vietnam. Essential determinants for implementation success were (1) adequate knowledge of the local context and embedding the programme into it (using existing health infrastructures), (2) collaborating with local influential stakeholders and motivating communities to actively participate and (3) flexibility throughout the process.

Other cascading train-the-trainer awareness programmes for lung health have previously demonstrated to be feasible in LMICs^40,41^. However, these studies mainly focussed on tobacco as a risk factor to lung health, while the need to address HAP is increasingly recognised^42^. Interestingly, these other programmes reported several essential factors of the implementation strategy comparable to those we had identified. Where we identified engaging influential and knowledgeable stakeholders, an Indian awareness programme on tobacco similarly defined the involvement of local role models (teachers) and leadership engagement (support from the school principals) as crucial^40^. Where we identified motivating the community, a PALSA study on CRD guidelines in South Africa reported actively involving participants in the delivery of the intervention^41^. Costs of these programmes were not reported, so cannot be compared. Both studies also reported the importance of compatibility of the intervention and implementation strategy with the local context, although they did not specifically emphasise the importance of embedding the programme into the local health infrastructures. A large overview of reviews on CHW programmes published in the *Lancet Global Health* in 2018 reported this embedment as a key recommendation for implementation success^43^.

We achieved statistically significant knowledge increases among all groups in both countries. The larger knowledge increase in Kyrgyzstan compared to Vietnam could be due to the lower baseline knowledge in Kyrgyzstan. Vietnam has had a longer tradition of patient education and patient self-management (and has established patient groups already decades ago). This may imply that awareness programmes could cover more advanced content in countries like Vietnam. Besides a higher increase in knowledge, also the acceptability and adequate use of cookstoves were higher in Kyrgyzstan compared to Vietnam after the awareness programme. This may indicate, in line with literature, that better knowledge on the risks of HAP to lung health is associated with higher success of clean cooking programmes^15,16^. Notably, rates for adequate adoption of the stoves were substantially higher in Kyrgyzstan compared to stove adoption rates from other studies. Adoption rates are often not reported in clean cookstove studies; if they are, it is commonly mentioned the rates are ‘strikingly low’, ‘disappointing’, or around 4–10%^11,44^. However, stove stacking occurred substantially more frequently in Vietnam in our study, suggesting that, besides knowledge, other causes also contribute to inadequate clean cooking practices. For example, characteristics of the stove are known to influence implementation success^15,16^; Vietnamese participants in the FRESH AIR stove programme considered their stove too small and continued to use their old one concurrently^39^. Hence, with many factors contributing to the adequate use of improved stoves, programme implementation should ideally go hand in hand with all favourable factors, such as favourable market developments and policies^15,16^. This gives this cascading train-the-trainer programme a particularly powerful potential when applied by policy makers, health workers and communities together, because then all different factors can be addressed simultaneously.

This study both aligns with the recent WHO guideline that emphasises on the role of CHWs in the prevention and treatment of (non-)communicable diseases^35^ and responds to the call to enhance focus on contexts during implementation^45,46^. Furthermore, we systematically applied and evaluated a uniform programme design in two completely different settings, enabling us to assess its wider applicability. This approach addresses the challenge of inconsistency in methodology and implementation assessment between training programmes for CHWs^47^. Another strength is the action research approach involving the whole system (from Ministry of Health to community), while generating real-world evidence. For example, the district health officers appointed the first HCWs to be trained. They supposedly selected the most capable and motivated HCWs, which is precisely what would happen in a non-study setting. Such an approach reduces selection bias and potential underestimations of the programme’s effect. Furthermore, the focus on implementation (fidelity) and its context—knowing what is ‘in the black box’—combined with effectiveness enabled us to relate the observed effect to the intervention with more confidence^48,49^. We are also among the few community-based implementation studies that included programme costs as an outcome^50^. The cascading train-the-trainer approach is designed to continue programme activities after the initial project has ended, thus contributing to the development of a sustainable system that builds knowledge and capacity among health workers and raises awareness in communities. As a limitation, our budget did not allow for observation of all implementation activities in vivo (precise number of delivered sessions, number of participants reached, etc.). Therefore, we relied on health workers’ self-reported implementation integrity. Social desirability might have tempted workers to over-report their implementation efforts^51^, possibly leading to an overestimation of fidelity. However, the number of completed knowledge questionnaires allowed us to triangulate and confirm the self-reported number of HCWs and CHWs trained and provide us with a minimum number of trained community members. Furthermore, although the effect was assessed at multiple levels in this study, each had its limitation. Validated questionnaires assessing knowledge about the risks of biomass and tobacco smoke did not exist to our knowledge. We therefore developed these questionnaires ourselves. In addition, the results from the questionnaires could be subject to selection bias. Also, although acceptability of the stoves was very high in both countries and stove stacking was particularly low in Kyrgyzstan^39^, we were unable to conclude whether these longer-term outcomes were causally related to the awareness programme. Many other factors are associated with adequate stove use^15^ and there was no control group. Tobacco-related behaviour change was not measured. Also, the financial barrier for behaviour change was less prominent in our study as the people received a small compensation for study participation (the price of the cheapest stove option in Vietnam or a stove donated by the World Bank in Kyrgyzstan). Therefore, conclusions on indications for effectiveness should be interpreted with caution.

Exposure to HAP and tobacco smoke continues to place a high burden on LMICs, not only through CRD but also through stroke, cardiovascular disease, ischaemic heart disease, pneumonia and lung cancer^42,52^. Beyond the health burden, there is a substantial socioeconomic burden of CRD in LMICs^53^. Effectiveness of previous lung health programmes is often hampered by implementation failure, further draining resource potential from already resource-limited settings and leading to poor health outcomes^11^. By demonstrating a feasible, acceptable and effective translation of an awareness programme in Uganda to two completely different settings—in Kyrgyzstan and Vietnam—we provide a potential guide for universal translation to other settings. The programme can be implemented on itself or, as applies to our FRESH AIR project, be an excellent starting point to prepare for smoking cessation programmes^54^ or clean cooking interventions^39^. This same implementation strategy of the programme could also be used to address other relevant health topics beyond lung health. We recommend to establish a relation with the community before implementing an awareness programme, for example by conducting a rapid assessment^55^ of the local context first. This will help to address the identified essential determinants for implementation success (adequate knowledge of the local context and embedding the programme into it, collaborating with local influential stakeholders and motivating communities to actively participate and flexibility).

To conclude, contextually translating a train-the-trainer awareness programme from Uganda to Kyrgyzstan and Vietnam, and potentially other low-resource settings, can be feasible, acceptable and effective for increasing awareness on lung health and its risk factors. Increased awareness empowers communities to take action to reduce exposure to biomass and tobacco smoke, which can ultimately lead to better lung health in low-resource settings.

## Methods

### Study design

This prospective implementation study was conducted between 2016 and 2018 within the FRESH AIR research project^56^. Reporting of this study was guided by the Standards for Reporting Implementation Studies (Supplementary Methods)^57^. The programme itself and the implementation strategy are detailed in Box 1, and the programme’s design is detailed in Fig. 1.

### Setting

We purposively selected Kyrgyzstan and Vietnam, as they represented two distinct low-resource settings with a high prevalence of CRDs and exposure to biomass and tobacco smoke^31,58^. In the highlands of Kyrgyzstan, >95% of households use wood or dung as their main fuel for their stoves (for cooking and heating); in the lowlands, approximately 30% use wood or coal^31,39^. Tobacco consumption is 26% (50% for men, 4% for women)^59^. In the Long An province of Vietnam, 75% of the households use solid fuels (65% use wood) for cooking^39^. Their tobacco consumption is 23% (47% for men, 1% for women)^59^. Pre-FRESH AIR fieldwork^31,60^ had revealed poor awareness on CRD in these countries. The exact settings were based on opportunity and the relationship already established with communities during earlier work. Further information on the settings is detailed in Supplementary Methods.

### Study population

Any HCW, CHW and community member was eligible to participate in the programme; there were no additional inclusion or exclusion criteria. The group of HCWs to initiate the train-the-trainer cascade was selected with help from locally influential stakeholders with expert knowledge of the context, such as district health officers. These HCWs then conveniently selected other HCWs or CHWs, usually within their vicinity. Subsequently, the CHWs trained (almost all) community members living in their village.

### Outcomes

We considered translation of the programme ‘feasible’ when it could be implemented with reasonable effort, budget and time and ‘acceptable’ if those delivering or receiving the programme responded emotionally and cognitively collaborative^61^. ‘Fidelity’ was considered to be high if the steps in programme were adhered to as intended (Fig. 1). Effectiveness was assessed at multiple levels; the immediate effect on CRD-related awareness (psychological capability in the COM-B) was assessed by knowledge questionnaires. The longer-term effect was expressed in degree of acceptability of improved stoves distributed in a subsequent FRESH AIR programme and behaviour (adequate use of the stoves)^39^. In this latter programme, households could select a locally manufactured improved cookstove/heater that they considered most suitable.

### Data collection and instruments

Data on the feasibility and acceptability of the programme, and lessons learned, were collected during face-to-face and online discussions throughout the entire implementation process. We discussed these topics until consensus was reached. The short-term effectiveness was assessed by a questionnaire for HCWs and one for both CHWs and community members. All HCWs and CHWs were invited to fill out the questionnaires as part of the training. Questionnaires contained several true/false/I-don’t-know statements relating to the programme’s content (Supplementary Methods). They were filled out before and after the training. Respondents were instructed to choose ‘true’/‘false’ when confident about an answer and to choose ‘I-don’t-know’ otherwise. The questionnaires were adapted according to lessons learned in Uganda^32^. They were translated to Russian and Vietnamese, respectively, back-translated to English, compared with the original versions and tailored accordingly. Acceptability and adequate use of improved stoves of the subsequent FRESH AIR programme were assessed by questionnaires and observations of stove stacking, respectively.

### Analysis

Feasibility and acceptability of the programme, and lessons learned, were qualitatively analysed, guided by the *modified Conceptual Framework for Implementation Fidelity*^62,63^. This framework focusses on adherence to complex health interventions, potential moderators and identifying ‘essential components’ for achieving the intended outcome (Table 2, left column). Effectiveness on awareness was determined by changes in people’s mean score on the pre- and post-training knowledge questionnaire, analysed by the Wilcoxon signed-rank tests (IBM SPSS Statistics version 25, Armonk, NY, USA). *P* values <0.05 were considered statistically significant. Indications for longer-term behavioural effectiveness (acceptability and adequate use of improved stoves) were calculated using descriptive statistics.

### Sample size and selection

We pragmatically aimed for 400 pre- and post-training community questionnaires. This number was chosen based on the maximum number of households that the budget allowed. Community members were randomly invited, stratified by gender, by the CHWs who gave the training. For the effect on acceptability and adequate use, 20 households in Kyrgyzstan and 76 in Vietnam were randomly invited in the stove programme.

### Ethics

The study complied with all ethical regulations and was approved by the research ethical review board of the University of Medicine and Pharmacy in Ho Chi Minh, Vietnam (188/DHYD-HD;06/27/2016) and the National Center of Cardiology and Internal Medicine Ethics Committee in Bishkek, Kyrgyzstan (5;03/03/2016). All participants with an improved stove provided written, informed consent before enrolment in the study. In case of illiteracy, the information was read to the participant and a thumb-print was provided instead. Other activities were within existing job descriptions (CHWs and HCWs) or regarded the attendance of routine educational activities upon personal initiative (community members).

### Reflexivity

Our team was diverse in terms of gender, age, professional background and nationality, contributing to diverse perspectives and richer data. To avoid hierarchy being at play, we emphasised that every person’s input during evaluations was equally valuable.

### Reporting summary

Further information on research design is available in the Nature Research Reporting Summary linked to this article.

## Supplementary information

Supplementary Information

Reporting Summary

## Data Availability

All data and meta-data will be available within a reasonable timeframe upon reasonable request.
