# Publisher Correction: Implementing a context-driven awareness programme addressing household air pollution and tobacco: a FRESH AIR study

**DOI:** 10.1038/s41533-020-00208-6

**Published:** 2020-10-20

**Authors:** Evelyn A. Brakema, Frederik A. van Gemert, Sian Williams, Talant Sooronbaev, Berik Emilov, Maamed Mademilov, Aizhamal Tabyshova, Pham Le An, Nguyen Nhat Quynh, Le Huynh Thi Cam Hong, Tran Ngoc Dang, Rianne M. J. J. van der Kleij, Niels H. Chavannes, Corina de Jong, The FRESH AIR collaborators, Marilena Anastasaki, Azamat Akylbekov, Andy Barton, Antonios Bertsias, Pham Duong Uyen Binh, Job F. M. van Boven, Dennis Burges, Lucy Cartwright, Vasiliki E. Chatzea, Liza Cragg, Ilyas Dautov, Irene Ferarrio, Ben Hedrick, Nick Hopkinson, Elvira Isaeva, Rupert Jones, Sanne van Kampen, Winceslaus Katagira, Jesper Kjærgaard, Janwillem Kocks, Le Thi Tuyet Lan, Tran Thanh Duv Linh, Christos Lionis, Kim Xuan Loan, Andy McEwen, Patrick Musinguzi, Rebecca Nantanda, Grace Ndeezi, Sophia Papadakis, Hilary Pinnock, Jillian Pooler, Charlotte C. Poot, Maarten J. Postma, Anja Poulsen, Pippa Powell, Susanne Reventlow, Dimitra Sifaki-Pistolla, Sally Singh, Jaime Correia de Sousa, James Stout, Marianne Stubbe Østergaard, Ioanna Tsiligianni, Tran Diep Tuan, James Tumwine, Le Thanh Van, Nguyen Nhu Vinh, Simon Walusimbi, Louise Warren

**Affiliations:** 1 Department of Public Health and Primary Care, Leiden University Medical Center, Leiden, The Netherlands; 2 Groningen Research Institute for Asthma and COPD (GRIAC) & Department of General Practice & Elderly Care Medicine, University of Groningen, University Medical Center Groningen, Groningen, The Netherlands; 3 Makerere University Lung Institute (MLI), Mulago Hospital, Kampala, Uganda; 4 International Primary Care Respiratory Group (IPCRG), London, UK; 5 Pulmonary Department, National Center of Cardiology and Internal Medicine, Bishkek, Kyrgyzstan; 6 Center of Training Family Medicine, University of Medicine and Pharmacy, Ho Chi Minh City, Vietnam; 7 Clinic of Social and Family Medicine, School of Medicine, University of Crete, Heraklion, Greece; 8 Faculty of Medicine and Dentistry, University of Plymouth, Plymouth, UK; 9 University of Groningen, University Medical Center Groningen, Groningen, The Netherlands; 10 Department of Pediatrics, University of Washington School of Medicine, Seattle, WA USA; 11 European Lung Foundation, Sheffield, UK; 12 Imperial College London, London, UK; 13 The Research Unit for General Practice and Section of General Practice, Department of Public Health, Copenhagen University, Copenhagen, Denmark; 14 Global Health Unit, The Department of Paediatrics and Adolescent Health, Juliane Marie Center, Copenhagen University Hospital “Rigshospitalet”, Copenhagen, Denmark; 15 National Centre for Smoking Cessation and Training, Dorchester, UK; 16 University of Edinburgh, Usher Institute of Population Health Sciences and Informatics, Edinburgh, UK; 17 Coventry University, Coventry, UK; 18 University of Minho, School of Medicine, Braga, Portugal

**Keywords:** Disease prevention, Patient education, Respiratory tract diseases, Public health, Translational research

Correction to: *npj Primary Care Respiratory Medicine* 10.1038/s41533-020-00201-z, published online 6 October 2020

In the original version of this Article, Table 2 was formatted in such a way that made it difficult to interpret. This has now been corrected in the PDF and HTML versions of the Article.

