# Supplementary material for: Implementing a context-driven awareness programme addressing household air pollution and tobacco: a FRESH AIR study
Source: NPJ Prim Care Respir Med. 2020 Oct 6;30:42. doi: 10.1038/s41533-020-00201-z (PMC7538921; doi:10.1038/s41533-020-00201-z)
Supplement: Supplementary file 1 — Supplementary Information [file 41533_2020_201_MOESM1_ESM.pdf]

SUPPLEMENTARY INFORMATION

Implementing a context-driven awareness programme addressing household air pollution and tobacco – a FRESH AIR study

Evelyn Brakema* & Frederik van Gemert*, Sian Williams, Talant Sooronbaev, Berik Emilov, Maamed Mademilov, Aizhamal Tabyshova, Pham Le An, Nguyen Nhat Quynh, Le Huynh Thi Cam Hong, Tran Ngoc Dang, Rianne van der Kleij, Niels Chavannes and Corina de Jong, on behalf of the FRESH AIR collaborators

CONTENT

Supplementary Methods:

- **Themes taught during the three-day workshop for healthcare workers *(COM-B element predominantly addressed)* (P2)**
- **Input for development of the implementation strategy (P3)**
- **Content of the flip-overs (P4)**
- **Knowledge questionnaires (P5)**
- **Background on the study settings (P6)**
- **Standards for Reporting Implementation Studies: the StaRI checklist for completion (P7)**

Supplementary Results:

- **Results of Knowledge Questionnaires (P10)**

Themes taught during the three-day workshop for healthcare workers
(COM-B element predominantly addressed)

COM-B model = capability, opportunity, motivation – behaviour.

- **Introduction to lung health**
  Capability – Psychological
    - Growth of the lungs, how lungs work, lung defence mechanisms, important issues before birth, during childhood and adulthood
- **What is COPD?**
  Capability – Psychological
    - Definition, pathology, symptoms, risk factors, assessment (spirometry), exacerbation, treatment (pharmaceutical and non-pharmaceutical)
- **What is asthma?**
  Capability – Psychological
    - Definition, symptoms, risk factors, assessment (spirometry), exacerbation, treatment (pharmaceutical and non-pharmaceutical)
- **Major chronic lung diseases and risk factors**
  Capability – Psychological / Motivation – Reflective
    - Major risk factors such as tobacco smoke, household air pollution, ambient air pollution, occupational exposures, allergens, post-infectious diseases and TB
- **Health effects of tobacco smoking**
  Capability – Psychological / Motivation – Reflective
    - Cardiovascular diseases, respiratory diseases, cancer, women and tobacco, pregnancy, second-hand smoke (perinatal and child health as a reason to stop smoking), tobacco and diabetes, TB and HIV, what happens when you quit
- **Health effects of household air pollution**
  Capability – Psychological / Motivation – Reflective
    - Adults (COPD, lung infection, TB infection, lung cancer, cardiovascular diseases, cataract, asthma, exacerbations), children (pneumonia, stunted growth, early childhood deaths, red eyes, running nose) and pregnancy (poor growth foetus, small born babies, perinatal mortality, nutritional deficiencies, pre-term delivery and poor lung maturation)
- **Infections of the lungs**
  Capability – Psychological / Motivation – Reflective
    - Factors promoting infections of the lungs, classified by site (upper and lower tract), by organism (viral, bacterial and fungal), and treatment
- **Intervention for smoking cessation**
  Capability – Psychological / Opportunity – Physical & Social / Motivation – Reflective
    - Why is it so difficult, biology of addiction and smoking cessation interventions such as brief interventions (3 A's and 5 A's), behavioural interventions and pharmacotherapy (what is available in concerned country)
- **Interventions to reduce household air pollution**
  Capability – Physical & Psychological / Opportunity – Physical & Social / Motivation – Reflective
    - Source of smoke (improving cooking devices, cleaner fuels), living environment (improve ventilation and kitchen design, placement of stove) and user (fuel drying, use of pot lids, good maintenance, reduction by avoiding smoke such as keeping children away from the fire)
- **Training methodologies and skills**
  Capability – Psychological / Opportunity – Social / Motivation – Reflective motivation
    - Characteristics of adult learning, creating a supportive environment, emphasizing personal benefit of training, active participation

- **Community mobilization techniques**
  Capability – Psychological / Opportunity – Physical & Social / Motivation – Reflective motivation
    - Involves activities that will have impact on community and individual knowledge, attitudes and practices

The final flip-over charts and posters from Uganda (and used as example for Kyrgyzstan and Vietnam) are available at: https://www.ipcrg.org/tobacco-dependence-uganda

Supplementary Table 1: Input for development of the implementation strategy

**Item**	**Key input for Kyrgyzstan**	**Key input for Vietnam**
Healthcare infrastructure *e.g. availability of health workers*	HCWs should be trained at all levels. Senior staff needed to be included as the healthcare system is hierarchical.<br><br>Training would be feasible within regular job description. However, due to rough terrains a long travel time was expected, so local team decided to omit workstream 2 and train the CHWs themselves. Training of communities could take place in public buildings or health stations. Also see main text Supplementary file 2.	Training would be an activity additional to the regular training of HCWs and CHWs. Therefore, financial compensation of training time was required. Trainings of communities could take place in health stations, committee offices, or houses of citizens (wherever convenient for the community). Also see main text Supplementary file 2.
Political infrastructure *e.g. working with the local government and village leaders*	Minister of Health endorsed programme and aimed for sustained implementation. Government officials were motivated to contribute. Village leaders would be open to participate.	Directors and officials welcomed programme, yet also had many other priorities which could potentially form a barrier for sustained implementation.
Programme acceptability<br><br>*e.g. cultural and social practice around tobacco smoking and biomass fuel use for cooking and heating*	Although harm of cigarette smoking is commonly understood, smoking prevalence is high among men. For women it is socially strongly disapproved to smoke but they suffer from second hand smoke exposure. Biomass fuel use is high, including during pregnancy. Health workers would be receptive for smoking cessation techniques.	Smoking is a manner to start social interactions between men. Smoking women are socially condemned, and women also suffer from second hand smoke exposure. Health workers would be receptive for smoking cessation techniques. Biomass fuel use is high, including during pregnancy.
Feasibility of reducing risk factors *e.g. the availability and affordability of alternative methods of cooking and heating*	Increased awareness could lead to alternative cooking methods, such increasing ventilation. Purchasing cleaner cooking devices might be feasible, yet is uncommon. Dung as fuel is often easily available for free. Usually there is no access to professional smoking cessation support. During this study period, a	Increased awareness could lead to alternative cooking methods, such as increasing ventilation. Purchasing cleaner cooking devices might be feasible, yet is uncommon. Usually there is no access to smoking cessation support. During this study period, a

	FRESH AIR smoking cessation intervention was concomitantly carried out.(39)	FRESH AIR smoking cessation intervention was concomitantly carried out.(39)
Additional message delivery *e.g. possible complementary delivery methods such as media, then taking into account media penetration, potential influence, and cost*	Many different media were considered influential and feasible, and the local team had experience with broadcasting health messages through TV. Radio and newspapers would be used too. Sending SMS text messages was perceived costly and time-consuming due to bureaucratic logistics in Uganda, so it was decided to omit this part of the awareness programme.	Media were considered influential, yet within the budget stakeholders and the local team considered a refresher course for trainers a priority above a media campaign. No SMS messages would be sent.

In Kyrgyzstan, after endorsement of the Minister of Health, the stakeholders were district health officers, government representatives, village leaders, communities, artists and health workers. In Vietnam, stakeholders were directors of district health services, a health centre, and a health education centre, and local healthcare workers.

Content of the flip-overs

Supplementary Table 2: Content of flip-overs

Lung health
• What are lungs?<br>• How lungs develop<br>• What can damage our lungs?<br>*People whose lungs can easily get damaged*<br>*Preventing lungs from damage/harm*
**Tobacco smoke**
• What are different ways tobacco is used?<br>• What are the effects of tobacco smoking?<br>• What can you do to reduce tobacco smoking?<br>• Benefits of quitting tobacco smoking<br>*What shall we do?*<br>*How can we find a solution to avoid exposure to tobacco smoke?*
**Biomass smoke**
• What is biomass smoke?<br>• What are the dangers of biomass smoke?<br>• How to control biomass smoke<br>• Short-term and long-term solutions

Knowledge questionnaires

For the healthcare workers (self-administered):
With these questions, we are trying to find out what people know about smoke we breathe in. So it is important for you to tell me if you don't know about the topic I ask about. We prefer that you don't guess the answers.
After each one, please circle the answer if it is true, false, of if you don't know for sure.

Supplementary Table 3: Knowledge questionnaire for healthcare workers

1	Biomass fuels are solid fuels made from grass, wood, crop residues, dung and charcoal used for cooking and heating. Our lungs can be damaged by breathing smoke during burning these biomass fuels.	□ **true** □ false □ I don't know
2	Smoking tobacco is only harmful to the smoker	□ true □ **false** □ I don't know
3	When a pregnant woman makes food using a traditional open fire with wood, this does not cause a higher chance of having a miscarriage	□ true □ **false** □ I don't know
4	Smoke from burning wood or dung is a major cause of lung infections like pneumonia among children under the age of 5 years	□ **true** □ false □ I don't know
5	Tobacco smoking increases the risk of developing diseases like tuberculosis (TB)	□ **true** □ false □ I don't know
6	Chronic obstructive pulmonary disease (COPD) is also a lung disease which can be caused by tobacco smoke, but not from smoke caused by burning wood or dung for cooking and heating	□ true □ **false** □ I don't know
7	When a pregnant woman smokes tobacco, this can result in having smaller babies	□ **true** □ false □ I don't know
8	Cigarettes can cause cancer in all parts of the body	□ **true** □ false □ I don't know
9	Smoke from open fires burning wood or dung can cause cataract, an eye disease causing clouding of the eye's natural lens	□ **true** □ false □ I don't know
10	A smoker needs help in order to quit smoking	□ **true** □ false □ I don't know

Answers reflecting correct knowledge are in **bold**

For the community members (researcher-administered):
With these questions, we are trying to find out what people know about smoke we breathe in. So it is important for you to tell me if you don't know about the topic I ask about. We prefer that you don't guess the answers. Now I am going to read you a series of statements. After each one, please tell me if it is true, false, or if you don't know for sure.

Supplementary Table 4: Knowledge questionnaire for community health workers and the community

1	The lungs help us to live by breathing air and removing the wasted gas from our body	□ **true** □ false □ I don't know
2	Biomass fuels are solid fuels made from grass, wood, crop residues, dung and charcoal used for cooking and heating. Our lungs can be damaged by breathing smoke from biomass fuels	□ **true** □ false □ I don't know
3	Smoking tobacco is only harmful to the smoker	□ true □ **false** □ I don't know
4	When a pregnant woman makes food using a traditional open fire with wood, this does not cause any problem during pregnancy	□ true □ **false** □ I don't know
5	Smoke from burning wood or dung is a major cause of lung infections like pneumonia among children under the age of 5 years	□ **true** □ false □ I don't know

Answers reflecting correct knowledge are in **bold**

Background on the study settings

Kyrgyzstan: The communities covered in Chui (700 m above sea level; 790,000 inhabitants) and Naryn district (2000-3500 m; 245,000 inhabitants) both have one regional hospital and several smaller territorial hospitals.[1] General practitioners work with nurses in Family Medicine Centres. Smaller villages usually have a health centre staffed by a nurse. CHWs in each village form a *village health committee*. They are chosen by the community and do not receive a salary.

Vietnam: The rural Can Giuoc district (180,000 inhabitants) is located in the Long An province south of Ho Chi Minh City.[2] There is one referral hospital for the whole district. Primary healthcare services are available in health centres (locally known as 'wards') which are generally staffed by clinical officers and nurses. CHWs form local *health volunteer teams*, who receive a compensation for the hours worked.

[1] National Statistical Committee of the Kyrgyz Republic. Population - 2018-жылдын 1-январына карата Кыргыз Республикасынын калкынын саны (The population of the Kyrgyz Republic as of January 1, 2018). 2018. Available at: http://stat.kg/en/. Accessed Nov 25, 2018.
[2] General Statistics Office of Vietnam. Statistics – Population and Employment. Available at: https://gso.gov.vn/Default_en.aspx?tabid=766. Accessed Nov 25, 2018.

Supplementary Standards for Reporting Implementation Studies: the StaRI checklist for completion

The StaRI standard should be referenced as: Pinnock H, Barwick M, Carpenter C, Eldridge S, Grandes G, Griffiths CJ, Rycroft-Malone J, Meissner P, Murray E, Patel A, Sheikh A, Taylor SJC for the StaRI Group. Standards for Reporting Implementation Studies (StaRI) statement. *BMJ* 2017;356:i6795

The detailed Explanation and Elaboration document, which provides the rationale and exemplar text for all these items is: Pinnock H, Barwick M, Carpenter C, Eldridge S, Grandes G, Griffiths C, Rycroft-Malone J, Meissner P, Murray E, Patel A, Sheikh A, Taylor S, for the StaRI group. Standards for Reporting Implementation Studies (StaRI). Explanation and Elaboration document. *BMJ Open* 2017 2017;7:e013318

Notes: A key concept of the StaRI standards is the dual strands of describing, on the one hand, the implementation strategy and, on the other, the clinical, healthcare, or public health intervention that is being implemented. These strands are represented as two columns in the checklist.

The primary focus of implementation science is the implementation strategy (column 1) and the expectation is that this will always be completed.	The evidence about the impact of the intervention on the targeted population should always be considered (column 2) and either health outcomes reported or robust evidence cited to support a known beneficial effect of the intervention on the health of individuals or populations.

The StaRI standardsrefers to the broad range of study designs employed in implementation science. Authors should refer to other reporting standards for advice on reporting specific methodological features. Conversely, whilst all items are worthy of consideration, not all items will be applicable to, or feasible within every study.

Supplementary Table 5

**Checklist item**		**Reported on page #**	**Implementation Strategy**	**Reported on page #**	**Intervention**
			"Implementation strategy" refers to how the intervention was implemented		"Intervention" refers to the healthcare or public health intervention that is being implemented.
**Title and abstract**					
Title	**1**	1	Identification as an implementation study, and description of the methodology in the title and/or keywords		
Abstract	**2**	2	Identification as an implementation study, including a description of the implementation strategy to be tested, the evidence-based intervention being implemented, and defining the key implementation and health outcomes.		
**Introduction**					
Introduction	**3**	3	Description of the problem, challenge or deficiency in healthcare or public health that the intervention being implemented aims to address.		

Rationale	**4**	3	The scientific background and rationale for the implementation strategy (including any underpinning theory/framework/model, how it is expected to achieve its effects and any pilot work).	3	The scientific background and rationale for the intervention being implemented (including evidence about its effectiveness and how it is expected to achieve its effects).
Aims and objectives	**5**	3	The aims of the study, differentiating between implementation objectives and any intervention objectives.		
**Methods: description**					
Design	**6**	10-12	The design and key features of the evaluation, (cross referencing to any appropriate methodology reporting standards) and any changes to study protocol, with reasons		
Context	**7**	11, Supplementary file P 5	The context in which the intervention was implemented. (Consider social, economic, policy, healthcare, organisational barriers and facilitators that might influence implementation elsewhere).		
Targeted 'sites'	**8**	11	The characteristics of the targeted 'site(s)' (e.g locations/personnel/resources etc.) for implementation and any eligibility criteria.	11	The population targeted by the intervention and any eligibility criteria.
Description	**9**	4	A description of the implementation strategy	4	A description of the intervention
Sub-groups	**10**	n/a	Any sub-groups recruited for additional research tasks, and/or nested studies are described		
**Methods: evaluation**					
Outcomes	**11**	11,12	Defined pre-specified primary and other outcome(s) of the implementation strategy, and how they were assessed. Document any pre-determined targets	11,12	Defined pre-specified primary and other outcome(s) of the intervention (if assessed), and how they were assessed. Document any pre-determined targets
Process evaluation	**12**	12	Process evaluation objectives and outcomes related to the mechanism by which the strategy is expected to work		
Economic evaluation	**13**	12	Methods for resource use, costs, economic outcomes and analysis for the implementation strategy	12	Methods for resource use, costs, economic outcomes and analysis for the intervention
Sample size	**14**	12	Rationale for sample sizes (including sample size calculations, budgetary constraints, practical considerations, data saturation, as appropriate)		
Analysis	**15**	12	Methods of analysis (with reasons for that choice)		

Sub-group analyses	**16**	n/a	Any a priori sub-group analyses (e.g. between different sites in a multicentre study, different clinical or demographic populations), and sub-groups recruited to specific nested research tasks		
**Results**					
Characteristics	**17**	5,6	Proportion recruited and characteristics of the recipient population for the implementation strategy	5,6	Proportion recruited and characteristics (if appropriate) of the recipient population for the intervention
Outcomes	**18**	3-8	Primary and other outcome(s) of the implementation strategy	3-8	Primary and other outcome(s) of the Intervention (if assessed)
Process outcomes	**19**	3-8	Process data related to the implementation strategy mapped to the mechanism by which the strategy is expected to work		
Economic evaluation	**20**	7	Resource use, costs, economic outcomes and analysis for the implementation strategy	7	Resource use, costs, economic outcomes and analysis for the intervention
Sub-group analyses	**21**	n/a	Representativeness and outcomes of subgroups including those recruited to specific research tasks		
Fidelity/ adaptation	**22**	3-8	Fidelity to implementation strategy as planned and adaptation to suit context and preferences	3-8	Fidelity to delivering the core components of intervention (where measured)
Contextual changes	**23**	3-8	Contextual changes (if any) which may have affected outcomes		
Harms	**24**	n/a	All important harms or unintended effects in each group		
**Discussion**					
Structured discussion	**25**	8-10	Summary of findings, strengths and limitations, comparisons with other studies, conclusions and implications		
Implications	**26**	8-10	Discussion of policy, practice and/or research implications of the implementation strategy (specifically including scalability)	8-10	Discussion of policy, practice and/or research implications of the intervention (specifically including sustainability)
**General**					
Statements	**27**	13	Include statement(s) on regulatory approvals (including, as appropriate, ethical approval, confidential use of routine data, governance approval), trial/study registration (availability of protocol), funding and conflicts of interest		

Results of Knowledge Questionnaires

Supplementary Table 6: Knowledge of health workers (social workers and community health workers) in Kyrgyzstan

N=90	Pre-test*	Post-test*
1. Biomass fuels are solid fuels made from grass, wood, crop residues, dung and charcoal used for cooking and heating. Our lungs can be damaged by breathing smoke during burning these biomass fuels.	64.2	100
2. Smoking tobacco is only harmful to the smoker	78.5	100
3. When a pregnant woman makes food using a traditional open fire with wood, this does not cause a higher chance of having a miscarriage	42.1	81.4
4. Smoke from burning wood or dung is a major cause of lung infections like pneumonia among children under the age of 5 years	53.5	95.2
5. Tobacco smoking increases the risk of developing diseases like tuberculosis (TB)	58.5	86.1
6. Chronic obstructive pulmonary disease (COPD) is also a lung disease which can be caused by tobacco smoke, but not from smoke caused by burning wood or dung for cooking and heating	54.2	72.8
7. When a pregnant woman smokes tobacco, this can result in having smaller babies	85.7	93.9
8. Cigarettes can cause cancer in all parts of the body	64.2	85.7
9. Smoke from open fires burning wood or dung can cause cataract, and eye disease causing clouding of the eye's natural lens	51.4	92.7
10.A smoker needs help in order to quit smoking	96.4	100

*Percentage of people that answered the question reflecting correct knowledge.

Supplementary Table 7: Knowledge of communities in Kyrgyzstan

N=535	Pre-test*	Post-test*
The lungs help us to live by breathing air and removing the wasted gas from our body	90.7	97.7
Biomass fuels are solid fuels made from grass, wood, crop residues, dung and charcoal used for cooking or heating. Our lungs can be damaged by breathing smoke from biomass fuels.	49	91
Smoking tobacco is only harmful to the smoker	50.7	78.9
When a pregnant woman makes food using a traditional open fire with dung, this does not cause any problem during pregnancy	33.6	89
Smoke from burning wood or dung is a major cause of lung infections like pneumonia among children under the age of 5 years	45.3	91.3

*Percentage of people that answered the question reflecting correct knowledge.

Supplementary Table 8: Knowledge of healthcare workers in Vietnam

N=17	Pre-test*	Post-test*
1. Biomass fuels are solid fuels made from grass, wood, crop residues, dung and charcoal used for cooking and heating. Our lungs can be damaged by breathing smoke during burning these biomass fuels.	75	100
2. Smoking tobacco is only harmful to the smoker	93.7	100

3. When a pregnant woman makes food using a traditional open fire with wood, this does not cause a higher chance of having a miscarriage	56.3	93.8
4. Smoke from burning wood or dung is a major cause of lung infections like pneumonia among children under the age of 5 years	50.0	93.8
5. Tobacco smoking increases the risk of developing diseases like tuberculosis (TB)	100	100
6. Chronic obstructive pulmonary disease (COPD) is also a lung disease which can be caused by tobacco smoke, but not from smoke caused by burning wood or dung for cooking and heating	43.8	93.8
7. When a pregnant woman smokes tobacco, this can result in having smaller babies	85.7	93.9
8. Cigarettes can cause cancer in all parts of the body	68.8	93.8
9. Smoke from open fires burning wood or dung can cause cataract, and eye disease causing clouding of the eye's natural lens	56.2	87.5
10.A smoker needs help in order to quit smoking	100	100

*Percentage of people that answered the question reflecting correct knowledge.

Supplementary Table 9: Knowledge of community health workers in Vietnam

N=77	Pre-test*	Post-test*
The lungs help us to live by breathing air and removing the wasted gas from our body	93.2	100
Biomass fuels are solid fuels made from grass, wood, crop residues, dung and charcoal used for cooking or heating. Our lungs can be damaged by breathing smoke from biomass fuels.	98.7	100
Smoking tobacco is only harmful to the smoker	56.8	66.7
When a pregnant woman makes food using a traditional open fire with dung, this does not cause any problem during pregnancy	68.9	81.3
Smoke from burning wood or dung is a major cause of lung infections like pneumonia among children under the age of 5 years	82.4	96.0

*Percentage of people that answered the question reflecting correct knowledge.

Supplementary Table 10: Knowledge of communities in Vietnam

N=385	Pre-test*	Post-test*
The lungs help us to live by breathing air and removing the wasted gas from our body	85.7	97.1
Biomass fuels are solid fuels made from grass, wood, crop residues, dung and charcoal used for cooking or heating. Our lungs can be damaged by breathing smoke from biomass fuels.	89.6	96.9
Smoking tobacco is only harmful to the smoker	53.1	58.2
When a pregnant woman makes food using a traditional open fire with dung, this does not cause any problem during pregnancy	57.1	74.0
Smoke from burning wood or dung is a major cause of lung infections like pneumonia among children under the age of 5 years	85.4	91.9

*Percentage of people that answered the question reflecting correct knowledge.
