# Supplementary material for: Implementing a context-driven awareness programme addressing household air pollution and tobacco: a FRESH AIR study
Source: NPJ Prim Care Respir Med. 2020 Oct 6;30:42. doi: 10.1038/s41533-020-00201-z (PMC7538921; doi:10.1038/s41533-020-00201-z)
Supplement: Supplementary file 2 — Reporting Summary [file 41533_2020_201_MOESM2_ESM.pdf]

nature research

Corresponding author(s): Evelyn Brakema and Frederik van Gemert

Last updated by author(s): Aug 16, 2020

Reporting Summary

Nature Research wishes to improve the reproducibility of the work that we publish. This form provides structure for consistency and transparency in reporting. For further information on Nature Research policies, see our Editorial Policies and the Editorial Policy Checklist.

Statistics

For all statistical analyses, confirm that the following items are present in the figure legend, table legend, main text, or Methods section.

n/a	Confirmed	
☐	☒	The exact sample size (*n*) for each experimental group/condition, given as a discrete number and unit of measurement
☐	☒	A statement on whether measurements were taken from distinct samples or whether the same sample was measured repeatedly
☐	☒	The statistical test(s) used AND whether they are one- or two-sided<br>*Only common tests should be described solely by name; describe more complex techniques in the Methods section.*
☐	☒	A description of all covariates tested
☐	☒	A description of any assumptions or corrections, such as tests of normality and adjustment for multiple comparisons
☐	☒	A full description of the statistical parameters including central tendency (e.g. means) or other basic estimates (e.g. regression coefficient) AND variation (e.g. standard deviation) or associated estimates of uncertainty (e.g. confidence intervals)
☒	☐	For null hypothesis testing, the test statistic (e.g. *F*, *t*, *r*) with confidence intervals, effect sizes, degrees of freedom and *P* value noted<br>*Give P values as exact values whenever suitable.*
☒	☐	For Bayesian analysis, information on the choice of priors and Markov chain Monte Carlo settings
☒	☐	For hierarchical and complex designs, identification of the appropriate level for tests and full reporting of outcomes
☒	☐	Estimates of effect sizes (e.g. Cohen's *d*, Pearson's *r*), indicating how they were calculated

Our web collection on statistics for biologists contains articles on many of the points above.

Software and code

Policy information about availability of computer code

Data collection	We used REDCap (Research Electronic Data Capture) for facilitating a secure, web-based application for capturing research data
Data analysis	IBM SPSS Statistics version 25, Armonk, NY, USA

For manuscripts utilizing custom algorithms or software that are central to the research but not yet described in published literature, software must be made available to editors and reviewers. We strongly encourage code deposition in a community repository (e.g. GitHub). See the Nature Research guidelines for submitting code & software for further information.

Data

Policy information about availability of data

All manuscripts must include a data availability statement. This statement should provide the following information, where applicable:

- Accession codes, unique identifiers, or web links for publicly available datasets
- A list of figures that have associated raw data
- A description of any restrictions on data availability

All data and meta-data will be available within a reasonable timeframe upon reasonable request.

Field-specific reporting

Please select the one below that is the best fit for your research. If you are not sure, read the appropriate sections before making your selection.

☐ Life sciences ☒ Behavioural & social sciences ☐ Ecological, evolutionary & environmental sciences

For a reference copy of the document with all sections, see nature.com/documents/nr-reporting-summary-flat.pdf

Behavioural & social sciences study design

All studies must disclose on these points even when the disclosure is negative.

Study description	Prospective implementation study, mixed-methods
Research sample	Any Kyrgyz and Vietnamese HCW, CHW and community member was eligible to participate in the programme; there were no additional in- or exclusion criteria.
Sampling strategy	The group of HCWs to initiate the train-the-trainer cascade was selected with help from locally influential stakeholders with expert knowledge of the context, such as district health officers. These HCWs then conveniently selected other HCWs or CHWs, usually within their vicinity. Subsequently, the CHWs trained (almost all) community members living in their village.
Data collection	Data on the feasibility and acceptability of the programme, and lessons learned, were collected during face-to-face and online discussions throughout the entire implementation process. We discussed these topics until consensus was reached. The short-term effectiveness was assessed by a questionnaire for HCWs, and one for both CHWs and community members. All HCWs and CHWs were invited to fill out the questionnaires as part of the training. Questionnaires contained several true/false/I-don't-know statements (Appendix 4) relating to the programme's content (Appendix 2 and 3). They were filled out before and after the training. Respondents were instructed to choose 'yes'/'no' when confident about an answer, and to choose 'I-don't-know' otherwise. The questionnaires were adapted according to lessons learned in Uganda.(32) They were translated to Russian and Vietnamese respectively, back-translated to English, compared with the original versions, and tailored accordingly. Acceptability and adequate use of improved stoves of the subsequent FRESH AIR programme were assessed by questionnaires and observations of stove stacking respectively.
Timing	Between 2016 and 2018
Data exclusions	No data were excluded from the analyses
Non-participation	No participants dropped out/declined participation
Randomization	Participants were not allocated to certain groups

Reporting for specific materials, systems and methods

We require information from authors about some types of materials, experimental systems and methods used in many studies. Here, indicate whether each material, system or method listed is relevant to your study. If you are not sure if a list item applies to your research, read the appropriate section before selecting a response.

Materials & experimental systems

n/a	Involved in the study	
☒	☐	Antibodies
☒	☐	Eukaryotic cell lines
☒	☐	Palaeontology and archaeology
☒	☐	Animals and other organisms
☐	☒	Human research participants
☐	☒	Clinical data
☒	☐	Dual use research of concern

Methods

n/a	Involved in the study	
☒	☐	ChIP-seq
☒	☐	Flow cytometry
☒	☐	MRI-based neuroimaging

Human research participants

Policy information about studies involving human research participants

Population characteristics	See above
Recruitment	The group of HCWs to initiate the train-the-trainer cascade was selected with help from locally influential stakeholders with expert knowledge of the context, such as district health officers. These HCWs then conveniently selected other HCWs or CHWs, usually within their vicinity. Subsequently, the CHWs trained (almost all) community members living in their village.

Ethics oversight	The study was approved by the research ethical review board of the University of Medicine and Pharmacy in Ho Chi Minh, Vietnam (188/DHYD-HD;06/27/2016) and the National Center of Cardiology and Internal Medicine Ethics Committee in Bishkek, Kyrgyzstan (5;03/03/2016).

Note that full information on the approval of the study protocol must also be provided in the manuscript.

Clinical data

Policy information about clinical studies

All manuscripts should comply with the ICMJE guidelines for publication of clinical research and a completed CONSORT checklist must be included with all submissions.

Clinical trial registration	TRIAL ID NTR5759
Study protocol	http://www.trialregister.nl/trialreg/admin/rctsearch.asp?Term=23332.
Data collection	We purposively selected Kyrgyzstan and Vietnam, see Appendix 2. Data were collected between 2016 and 2018.
Outcomes	We considered translation of the programme ‘feasible’ when it could be implemented with reasonable effort, budget and time, and ‘acceptable’ if those delivering or receiving the programme responded emotionally and cognitively collaborative.(61) ‘Fidelity’ was considered to be high if the steps in programme were adhered to as intended (Figure 1). Effectiveness was assessed at multiple levels; the immediate effect on CRD-related awareness (psychological capability in the COM-B) was assessed by knowledge questionnaires. The longer-term effect was expressed in degree of acceptability of improved stoves distributed in a subsequent FRESH AIR programme, and behaviour (adequate use of the stoves).(41) In this latter programme, households could select a locally manufactured improved cookstove/heater that they considered most suitable.
